# Bilateral Trapezoid-Like Scrotal Flaps for Reconstruction of Partial Scrotal Defects after Fournier's Gangrene

**DOI:** 10.1055/a-2875-2047

**Published:** 2026-07-06

**Authors:** Yusuf Alper Aytaç, Mustafa Canberk Gürbüz, Emrah Kağan Yaşar, Ceyhun Uzun, Murat Şahin Alagöz

**Affiliations:** 1 Department of Plastic, Reconstructive and Aesthetic Surgery 668971 Kocaeli City HospitalIzmitKocaeliTurkey; 2 Department of Plastic 52980 Reconstructive and Aesthetic Surgery, Kocaeli University HospitalİzmitKocaeliTurkey

**Keywords:** Fournier's gangrene, scrotal reconstruction, perineal reconstruction, scrotal flap, trapezoid-like flap

## Abstract

Perineal reconstruction in males can be challenging due to the complex anatomy and the potential for complications such as infection, hematoma, and wound dehiscence. In this retrospective case series, we present seven patients who underwent perineal reconstruction between 2020 and 2023 using bilateral scrotal flaps. All defects were secondary to debridement for Fournier's gangrene. In patients with defects involving up to 70% of the total scrotal surface and with sufficient intact scrotal tissue preserved superiorly and inferiorly, reconstruction was performed using bilateral trapezoid-like scrotal flaps based on perforators of the anterior and posterior scrotal arteries. The mean follow-up period was 6 months. Longer-term follow-up was not consistently available for all patients due to loss to follow-up. Nevertheless, selected cases were followed up for 1 year, as demonstrated in the clinical figures. Clinical outcomes were evaluated in seven patients enrolled in the study. The median operative time was 48 (43.5–53.5) minutes. The median length of hospital stay was 3 (2.5–4.0) days. Complete healing time was recorded as 19 (16.5–21.0) days. All flaps used for tissue coverage were successful. Among the observed complications, wound dehiscence was identified as the most prevalent, which occurred in three of seven patients (42.9%) and was managed successfully with local wound care and minor debridement. No partial or total flap loss was observed. Due to its short operating time, low donor site complication rate, and favorable tissue match, the trapezoid-like flap may represent a practical and reliable option in selected patients.

## Introduction

The perineum is a complex region of the body that contains many vital structures, including the urethra, anus, and genitals. Perineal reconstruction in males can be challenging due to this complexity and the potential for complications, such as infection, hemostatic complications, including hematoma, and wound dehiscence. Patients undergoing perineal reconstruction should be carefully selected and thoroughly evaluated to ensure the best possible outcomes. 1 Several techniques for perineal reconstruction in males are available, depending on the extent and location of the injury or defect. These techniques include local primary wound closure, split-thickness skin grafting, local advancement flaps, scrotal flaps, regional pedicled flaps, and free tissue transfer. 2 3 The choice of reconstructive method is primarily determined by the extent of tissue loss, the condition of the residual scrotal skin, and patient-related factors. When sufficient scrotal tissue is preserved, reconstructions based on local scrotal flaps are generally preferred, whereas skin grafts or distant flap reconstructions are typically reserved for more extensive defects. Within this reconstructive spectrum, the technique described in the present study represents a targeted local scrotal flap option for carefully selected patients with partial preservation of native scrotal tissue. 4 5 In this study, we present our cases that underwent perineal reconstruction by using bilateral trapezoid-like scrotal flaps. In contrast to conventional scrotal advancement or transposition flaps, the technique described in this study utilizes a bilateral trapezoid-like design that enables redirection of preserved superior scrotal tissue toward inferior defects. This configuration increases tissue excursion and reduces closure tension compared with linear advancement alone. Therefore, it may allow reconstruction of relatively larger partial scrotal defects while maintaining reliable vascularity and minimizing donor site morbidity.

## Idea

This study represents a retrospective case series describing a technical approach to partial scrotal reconstruction. Seven patients with scrotal defects, secondary to the debridement of Fournier's gangrene, who underwent scrotal reconstruction between the years 2020 and 2023, using a bilateral trapezoid-like scrotal flap technique, were reviewed. This study was approved by the Institutional Review Board (IRB approval number 202603). Written informed consent was obtained from all patients for participation in the study and for the use of their clinical photographs for scientific and publication purposes. A brief summary of the cases, associated comorbidities, and additional interventions performed is presented in Tables 1 and 2 . There are many procedures described for scrotal reconstruction. The technique presented herein, however, might be useful in only a particular group of patients with a particular type of scrotal defect. Patients were considered eligible for this technique only if at least 30% of the native scrotal skin and the proximal one-third of the scrotal raphe were preserved.

**Table 1:** Associated comorbidities in patients who were operated on with bilateral trapezoid-like flaps

Patient	Age (years)	Comorbidities	Smoking status
1	35	Diabetes mellitus	Yes
2	38	None	Yes
3	41	Diabetes mellitus	Yes
4	50	Diabetes mellitus	Yes
5	52	Diabetes mellitus, hypertension, colon cancer	Yes
6	57	None	Yes
7	57	Diabetes mellitus	Yes

**Table 2:** Defect size and postoperative complications of patients who were operated on with bilateral trapezoid-like flaps

Patient	Defect size	Postoperative complication	Intervention
1	60% of the scrotal surface	Wound dehiscence	Debridement and suturing
2	60% of scrotal surface	None	None
3	60% of scrotal surface	None	None
4	70% of scrotal surface	Wound dehiscence	Debridement and suturing
5	60% of scrotal surface	None	None
6	70% of scrotal surface	Wound dehiscence	Debridement and suturing
7	70% of scrotal surface	None	None

As a brief anatomy revisit will serve to remind us, the vascular support of the scrotal skin is mainly based on internal and external pudendal arteries and their branches, the posterior and anterior scrotal arteries, respectively. 6 Two trapezoid-like flaps, which are predominantly based on the perforators that arise from the anterior and posterior scrotal arteries, are planned. Both flaps are based on the scrotal raphe proximally and extend to the inguinal crease distally. Flaps are elevated just above the level of the testicles (see Figs. 1 and 2 ). Testicular structures are preserved, and two fasciocutaneous flaps are obtained. The flaps were transposed and adapted to their new locations, thereby facilitating the closure of the defects. All donor sites were primarily closed.

**Fig. 1:**
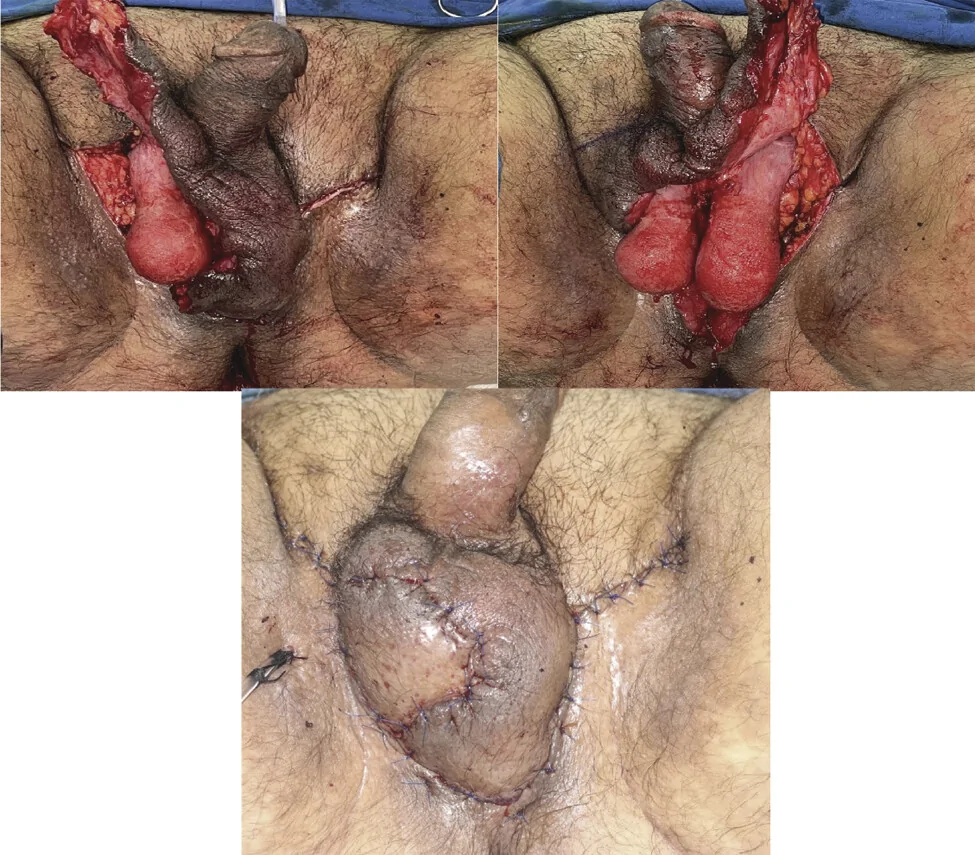
Perioperative appearance of a patient after elevation of the flaps in a bilateral trapezoid-like flaps procedure (above left and right) and immediate postoperative result.

**Fig. 2:**
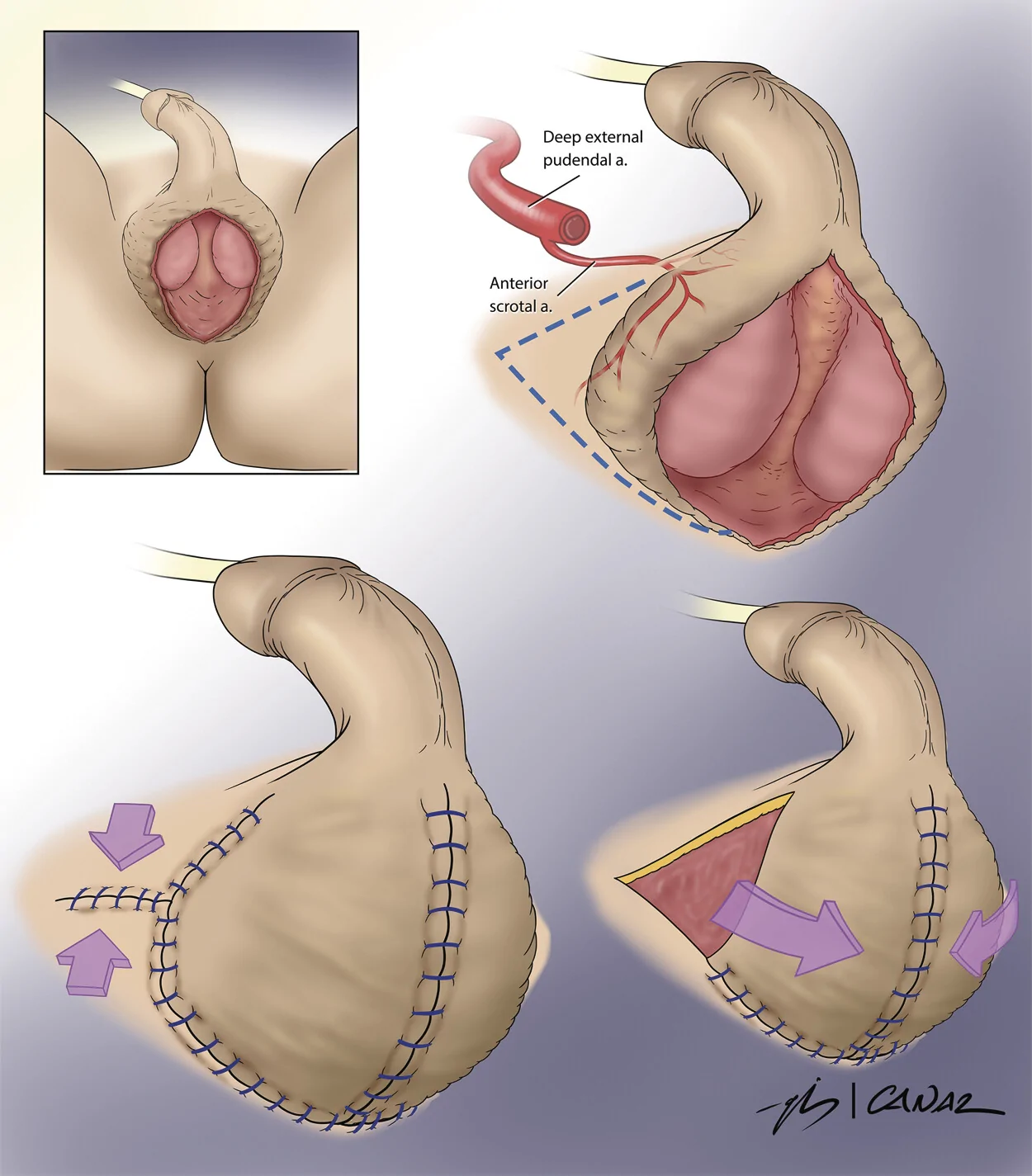
Schematic representation of our surgical planning for patients operated on with bilateral trapezoid-like flaps.

Clinical outcomes were evaluated in seven patients enrolled in the study. The median operative time was 48 (43.5–53.5) minutes. The median length of hospital stay was 3 (2.5–4.0) days. Complete healing time was recorded with a median of 19 (16.5–21.0) days.

Wound dehiscence occurred in three of seven patients (42.9%) and was the most common complication. All cases were managed successfully with local wound care and minor debridement. No partial or total flap loss was observed.

The bilateral trapezoid-like scrotal flaps described in this study are based on perforators arising from the anterior and posterior scrotal arteries, which have been shown to provide a reliable and consistent vascular supply to the scrotal skin. The rich and redundant vascular anatomy of the scrotum has been well-documented in the literature, with predictable perforators originating from both the external and internal pudendal systems. 6 In the present series, no preoperative imaging or intraoperative vascular mapping (such as Doppler ultrasonography or CT angiography) was routinely performed. Instead, flap design and elevation were based on established anatomical landmarks, including the scrotal raphe as the proximal flap base, the plane just superficial to the testicular structures, and limitation of flap extension within the inguinal crease, together with direct intraoperative assessment of flap perfusion. 6

Case examples are shown in Figs. 1 , 3 , and 4 .

**Fig. 3:**
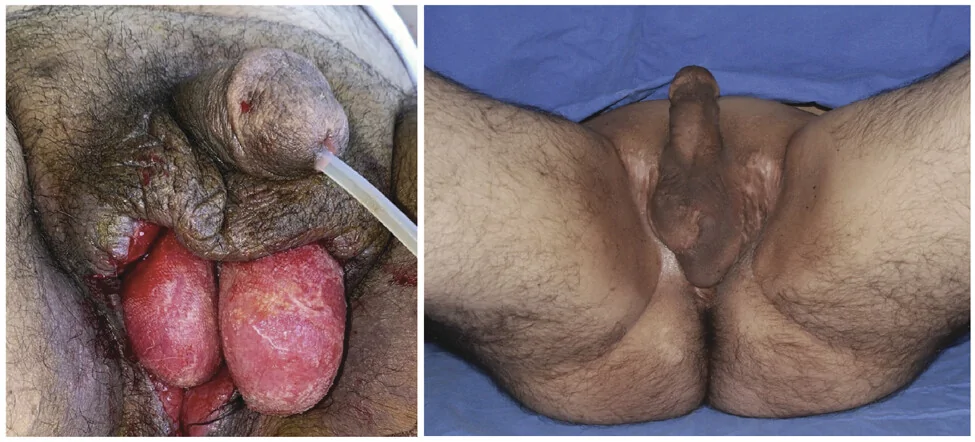
Preoperative (left) and late-term result 1-year postoperative (right) images are shown.

**Fig. 4:**
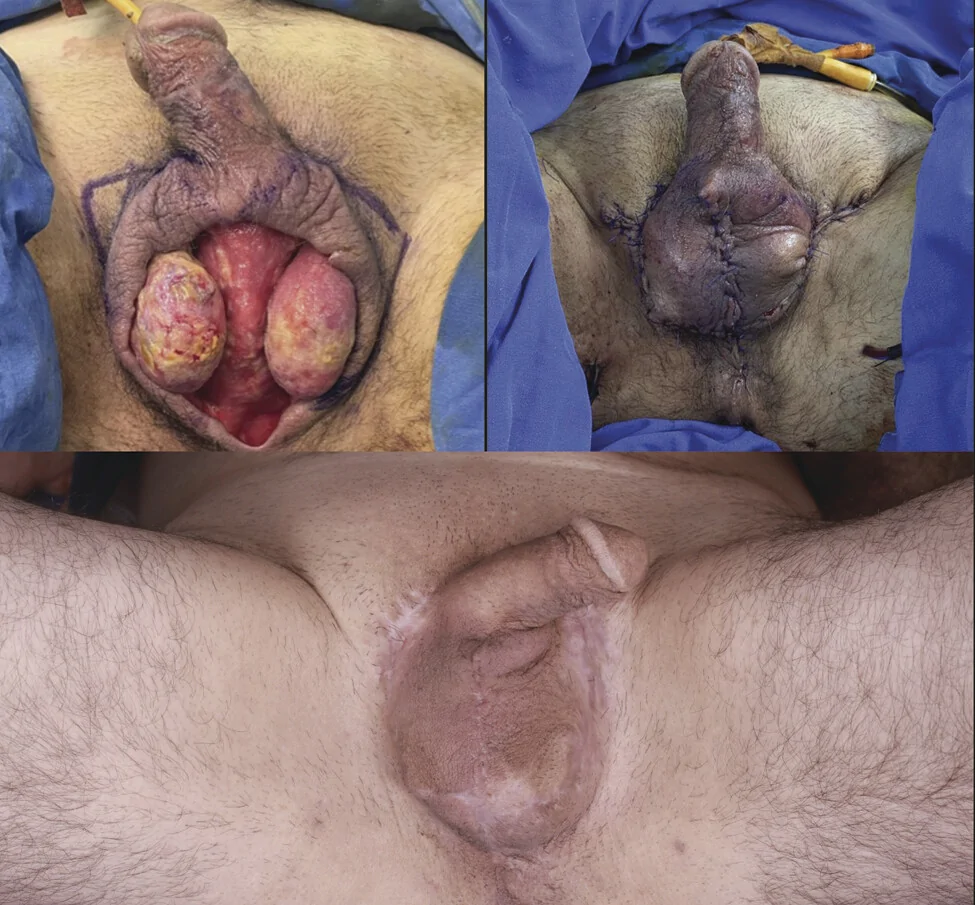
Perioperative (top left), early postoperative (top right), and late postoperative (1 year) appearance of a patient operated on using bilateral trapezoid-like flaps.

## Discussion

Scrotal defects can have various etiologies. Some of the most common causes of scrotal defects include trauma, infection, and inflammation. Infections, including sexually transmitted infections or bacterial infections, can also lead to scrotal defects. 7 8 9

Reconstructing the scrotum can be a complex process due to the desire for an aesthetically pleasing result and maintenance of testicular function, as well as difficulty with skin closure. 10

The technique used to reconstruct the scrotal area depends on various factors, such as the size and location of the defect, age of the patient, existing health conditions, and the surgeon's preference. 4 Delayed primary closure and split-thickness skin graft (STSG) are both safe and effective single-stage procedures that require a shorter hospital stay. 2 However, delayed primary closure is only suitable for scrotal defects that involve less than 50% of surface area, and STSG is not recommended if the tunica vaginalis is damaged. 11 Fasciocutaneous flaps offer an alternative option that provides well-vascularized soft tissue coverage in a single stage. 12 13

For larger defects, local pedicled muscle or fasciocutaneous flaps or free tissue transfer can be used. 14 Although there are many potential techniques that offer successful tissue coverage, unfortunately, most of these methods may present increased surgical difficulty and donor site morbidity. 15 Although the newly reconstructed scrotum has a similar appearance and temperature to the original, it lacks excess skin, and the testicles may remain in a lower position due to loss of cremasteric function. Tissue expanders are also a potential option for scrotal reconstruction. 16 A potential temporary solution for testicular coverage during reconstruction is moving the testes to the subcutaneous tissues of the upper thigh area. However, this approach has drawbacks, such as negative effects on sperm production, testicular shrinkage, and ongoing pain due to increased temperature in the area. 17

For smaller scrotal defects, scrotal advancement, transposition, and rotation flaps can be used to mobilize and cover the remaining tissue, resulting in a natural appearance, similar to the original scrotum. Por et al reported that to reconstruct scrotal defects with scrotal tissue itself, nearly one-third of scrotal skin remnants would be sufficient to reconstruct the entire scrotum. 18 Methods incorporating this type of reconstruction have a minimal impact on sperm production and no donor site morbidity, making them safe and simple with reduced hospital stay and costs. 19

The technique presented herein might be useful in only a particular group of patients with a particular type of scrotal defect. In all cases, more than 30% of the scrotal skin and the proximal one-third portion of the scrotal raphe remained intact, and our technique depended on harvesting two trapezoid-like flaps used for scrotal reconstruction. Unlike simple mobilization of residual scrotal tissue, the bilateral trapezoid-like flap design allows redirection of preserved superior scrotal skin toward the inferior defect rather than linear advancement alone. 1 2 This transposition-based movement increases tissue excursion and facilitates flap mobilization with reduced closure tension. As a result, larger partial scrotal defects can be reconstructed while achieving more effective testicular coverage compared with advancement-based techniques. All flaps demonstrated reliable perfusion without vascular compromise, supporting the anatomical consistency of these perforators in this selected patient cohort. Due to its short operating time and low donor site complication rate, this technique is preferred as one of the top options for this type of reconstructive surgery in our clinical practice.

As for the limitations, this technique is applicable only to patients with a specific type of defect, contingent upon the fulfillment of certain conditions. If these requirements are not met, alternative strategies must be considered. Due to the retrospective nature of the study and the emergency setting of Fournier's gangrene, objective patient-reported outcome measures and sexual function scores were not available. Although adequate testicular coverage was achieved in all patients, formal assessment of fertility, patient-reported outcomes, and cosmetic satisfaction was not performed. Additionally, follow-up duration was not uniform across all patients due to loss to follow-up, which may be related to socioeconomic factors. This should be considered when interpreting long-term outcomes. Nevertheless, selected cases were followed up for 1 year, as demonstrated in the clinical figures.

Perineal reconstruction is an important surgical procedure for restoring the structure and function of the perineum in males. With careful patient selection and appropriate surgical technique, perineal reconstruction can help to improve the quality of life and well-being of patients affected by perineal trauma or defects. The use of bilateral trapezoid-like flaps for reconstruction of scrotal defects after treatment for Fournier's allows for straightforward harvesting, ensuring a good match of the tissue and overall a good like-for-like reconstruction. Furthermore, the construction of flaps is constrained within the perineal region and does not extend beyond the inguinal crease or abdominal area. We believe that employing this technique yields favorable outcomes when reconstructing patients with this particular type of scrotal defect.
